# Retraction Note: Barbaloin: an amazing chemical from the ‘wonder plant’ with multidimensional pharmacological attributes

**DOI:** 10.1007/s00210-024-03064-0

**Published:** 2024-04-13

**Authors:** Shreya Sikdar Mitra, Mimosa Ghorai, Samapika Nandy, Nobendu Mukherjee, Manoj Kumar, Radha, Arabinda Ghosh, Niraj Kumar Jha, Jarosław Proćków, Abhijit Dey

**Affiliations:** 1 Department of Life Sciences, Presidency University, 86/1 College Street, Kolkata, 700073 West Bengal India; 2 Department of Health Sciences, Novel Global Community Educational Foundation, Hebersham, Australia; 3 Chemical and Biochemical Processing Division, ICAR-Central Institute for Research On Cotton Technology, Mumbai, 400019 Maharashtra India; 4 School of Biological and Environmental Sciences, Shoolini University of Biotechnology and Management Sciences, Solan, 173229 Himachal Pradesh India; 5 Department of Botany, Gauhati University, 781014 Guwahati, Assam India; 6 Department of Biotechnology, School of Engineering & Technology, Sharda University, Greater Noida, Uttar Pradesh 201310 India; 7 Department of Biotechnology Engineering and Food Technology, Chandigarh University, Mohali, 140413 Punjab India; 8 Department of Biotechnology, School of Applied & Life Sciences, Uttaranchal University, Dehradun, 248007 Uttarakhand India; 9 Department of Plant Biology, Institute of Environmental Biology, Wrocław University of Environmental and Life Sciences, Kożuchowska 5b, 51-631 Wrocław, Poland

**Retraction Note: Naunyn-Schmiedeberg’s Archives of Pharmacology (2022) 395:1525–1536**

10.1007/s00210-022-02294-4

The Editor-in-Chief has retracted this article following concerns that were raised after publication. Subsequent post-publication peer review has concluded that this article does not present a balanced review of the field: research results from other publications are overinterpreted, always in the sense of a positive presentation of the effects of the Aloe vera plant, and there is repeated presentation of the same facts. The Editor-in-Chief no longer has confidence in the conclusions drawn.

Shreya Sikdar Mitra, Mimosa Ghorai, Samapika Nandy, Nobendu Mukherjee, Manoj Kumar, Radha, Arabinda Ghosh, Niraj Kumar Jha, Jarosław Proćków and Abhijit Dey do not agree with this retraction.

